# European Portuguese Version of the User Satisfaction Evaluation Questionnaire (USEQ): Transcultural Adaptation and Validation Study

**DOI:** 10.2196/19245

**Published:** 2021-06-29

**Authors:** Célia Domingos, Patrício Soares Costa, Nadine Correia Santos, José Miguel Pêgo

**Affiliations:** 1 Life and Health Sciences Research Institute School of Medicine University of Minho Braga Portugal; 2 ICVS/3B's PT Government Associate Laboratory Braga Portugal; 3 iCognitus4ALL - IT Solutions Braga Portugal; 4 Clinical Academic Center - Braga Braga Portugal; 5 Faculty of Psychology and Education Sciences University of Porto Braga Portugal; 6 Associação Centro de Medicina Digital P5 Braga Portugal

**Keywords:** satisfaction, usability, reliability, validity, seniors, elderly, technology, wearables

## Abstract

**Background:**

Wearable activity trackers have the potential to encourage users to adopt healthier lifestyles by tracking daily health information. However, usability is a critical factor in technology adoption. Older adults may be more resistant to accepting novel technologies. Understanding the difficulties that older adults face when using activity trackers may be useful for implementing strategies to promote their use.

**Objective:**

The purpose of this study was to conduct a transcultural adaptation of the User Satisfaction Evaluation Questionnaire (USEQ) into European Portuguese and validate the adapted questionnaire. Additionally, we aimed to provide information about older adults’ satisfaction regarding the use of an activity tracker (Xiaomi Mi Band 2).

**Methods:**

The USEQ was translated following internationally accepted guidelines. The psychometric evaluation of the final version of the translated USEQ was assessed based on structural validity using exploratory and confirmatory factor analyses. Construct validity was examined using divergent and discriminant validity analysis, and internal consistency was evaluated using Cronbach α and McDonald ω coefficients.

**Results:**

A total of 110 older adults completed the questionnaire. Confirmatory factor analysis supported the conceptual unidimensionality of the USEQ (χ^2^_4_=7.313, *P*=.12, comparative fit index=0.973, Tucker-Lewis index=0.931, goodness of fit index=0.977, root mean square error of approximation=0.087, standardized root mean square residual=0.038). The internal consistency showed acceptable reliability (Cronbach α=.677, McDonald ω=0.722). Overall, 90% of the participants reported excellent satisfaction with the Xiaomi Mi Band 2.

**Conclusions:**

The findings support the use of this translated USEQ as a valid and reliable tool for measuring user satisfaction with wearable activity trackers in older adults, with psychometric properties consistent with the original version.

## Introduction

The use of mobile health (mHealth) technology has greatly increased over the past decade. These technologies enable health promotion and self-monitoring of health-related behaviors [1-3] and show potential in disease treatment and prevention in a cost-efficient and widely accessible manner [4]. Currently, mHealth covers a wide range of technologies, such as wearable devices and smartphone apps, for tracking different types of health-related data including physical activity [4,5].

Activity trackers are sensor-based wearable devices that automatically track and monitor various indicators of physical activity (eg, steps, calories burned, and distance traveled), with some also able to record heart rate and sleep measures [2,6-8]. The technology has the potential to help older adults with health self-management and self-efficacy by improving lifestyle behaviors and motivating compliance or attainment of daily activity goals [3,6,9]. Despite this potential, most older adults do not use activity trackers for their health-tracking needs. A possible explanation may be a matter of usability. Although it is a critical factor that can determine technology adoption, these devices have been mainly developed for a younger target group; thus, older adults may have difﬁculties due to usability barriers [2,9].

Published by the International Organization for Standardization, ISO-9241-11 defines usability in terms of effectiveness, efficiency, and user satisfaction rating of a product in a specific environment, by a specific user, for a specific purpose [10,11]. Accordingly, user satisfaction can be thought of as a usability component, but it cannot be evaluated in the same manner as efficiency and effectiveness. Satisfaction is the user’s attitude toward the system they use, affecting behavior intention for continuous or future use [12-14]. Moreover, satisfaction depends on how comfortable the user feels using the system [10,11,14,15]. Despite the importance of user satisfaction to ensure usability and the improved development of mHealth solutions, there is a gap in research assessing user satisfaction with mHealth [4].

Currently, there are a few validated and widely used questionnaires to collect targeted user feedback for the evaluation of a system’s usability. These include the System Usability Scale (SUS) [15], the Post-Study System Usability Questionnaire (PSSUQ) [10,16-18], and the Usefulness, Satisfaction and Ease of Use (USE) questionnaire [10,17,19]. Of these, only the SUS [20] and the USE [21] questionnaires are available in European Portuguese for generalized evaluation of usability. Regarding user satisfaction questionnaires, a study by Melin et al [4] presented the development of the mHealth Satisfaction Questionnaire, the Questionnaire for User Interaction Satisfaction (QUIS) [22], and the User Satisfaction Evaluation Questionnaire (USEQ). Nonetheless, despite this, there is no validated questionnaire for measuring user satisfaction with technologies available in European Portuguese.

Therefore, this study aimed to provide a valid questionnaire to speciﬁcally evaluate user satisfaction with technologies in Portuguese older adults. For this, the USEQ was selected since it is a short but comprehensive questionnaire with a reasonable number of questions; importantly, it is clear and easy to understand [23]. We also aimed to evaluate user satisfaction with the Xiaomi Mi Band 2 among older adults. Regardless of spoken language, and despite a growing number of studies on subjective experiences of user satisfaction with and the usability and usefulness of wearable technologies, only a few studies have focused on older adults [3,8,24]. Understanding whether a cohort of older adults is satisfied with these activity trackers is important to ensure devices can be successfully implemented in clinical and research settings [25].

## Methods

The study was divided into two phases. Phase 1 addressed the translation of the USEQ questionnaire into European Portuguese and its cross-cultural adaptation. Phase 2 involved the assessment of the USEQ psychometric properties and its validation in the new context.

### Translation and Cultural Adaptation of the USEQ

The USEQ is composed of 6 questions and uses a 5-point Likert scale for responses. The total score ranges from 6 (poor satisfaction) to 30 (excellent satisfaction). All questions are affirmative, except question 5, which is a negatively posed question. The numerical value of the affirmative questions is used to calculate the score. The negative question subtracts the numerical value of the response from 6 and then adds this result to the total score [23]. Since the USEQ was designed to evaluate user satisfaction with virtual rehabilitation systems, the questionnaire comprises one item that specifically measures the perceived usefulness of using the technology for rehabilitation. Thus, in this study, we adapted this item to include an item that could be applied to general-purpose systems or across different types of mHealth.

The adapted English version of the USEQ was culturally and linguistically adapted to European Portuguese after obtaining formal authorization from the original author (Gil-Gómez [23]). The process of translation followed the general guidelines provided by Lenz et al [26] and the World Health Organization [27]. Briefly, it comprised the following steps: forward translation, translation review and reconciliation of content, back translation, preliminary version, pretesting, and final version (Multimedia Appendix 1). In step 1 (forward translation), the USEQ questionnaire was translated to European Portuguese by two independent English-proficient translators, whose native language is European Portuguese. In step 2 (translation review and reconciliation of content), the independent translations were reviewed by both forward translators as well as by an independent team member. A reconciliation version of the document was obtained. In step 3 (back translation), the reconciled version was translated from European Portuguese into English by independent translators fluent in both languages, who were blinded to the original USEQ version of the document. The retroversion was done as a quality control step and to verify that both versions were equivalent. In step 4 (preliminary version), all team members performed a comparative analysis between the back-translation and original version. A preliminary version was prepared after items were reviewed and a consensus was reached. Finally, in step 5 (pretesting), the preliminary version of the document was tested in a pilot study with a sample of 20 independent and representative individuals selected from those who will be administered the final questionnaire. The individuals were asked to provide feedback on their understanding of the questions, the preferred use of alternative words for a given expression, terms deemed unacceptable or offensive, and their opinion of the questionnaire. The information collected was used to improve and develop the final version of the USEQ.

### Psychometric Properties and Validation of the USEQ

The psychometric validation of the final version of the USEQ was based on real data collection after the users’ experience with the wearable activity tracker. The analyses included assessment of structural validity, construct validity, and internal consistency.

To provide evidence of construct validity, the participants’ responses on the USEQ were correlated with the pre-existing instruments that measure similar concepts—the SUS and the technology acceptance model 3 (TAM3; convergent validity). For divergent validity, the participants’ responses on the USEQ were correlated with Mini-Mental State Examination (MMSE) scores [28]. Briefly, the SUS is the most widely used standardized questionnaire to measure perceived usability [10,15,29,30], while the TAM is the most applied theoretical model for evaluating or predicting users’ acceptance of new technologies [31]. Lastly, the MMSE is a widely accepted questionnaire to assess cognitive function in older adults [32].

### Participants and Data Collection

Following the application of inclusion and exclusion criteria, a total of 120 community-dwelling older adults (aged 64-75 years) from Northern Portugal were recruited to the study. The primary exclusion criteria were an inability to understand informed consent and neuropsychiatric and neurodegenerative disorders. Among the recruited participants, a final sample of 110 participants completed the usability test of the wearable activity tracker.

A baseline characterization was performed through a sociodemographic questionnaire and a standardized clinical interview. Moreover, since individual differences, including demographics, cognitive state, and emotional state inﬂuence individuals’ perceptions regarding the technology [33], we included a neuropsychological evaluation to obtain mood (Geriatric Depression Scale [GDS]) [34] and global cognitive profiles (MMSE) [35].

For usability testing, the Xiaomi Mi Band 2 was selected among several commercially available wearable activity trackers, since it is ergonomic, accessible, easy to operate, and offers the best price-quality ratio. The device provides general health monitoring, combining sensors that allow objective assessment of activity levels, heart rate, and sleep patterns [5,36,37]. The participants used a Xiaomi Mi Band 2 over 15 days while performing their normal daily activities. They were instructed to wear the activity tracker continuously. After concluding the usage testing period, participants were asked to provide information about their experience. This was attained through application of the USEQ [23] to evaluate user satisfaction, the TAM 3 [38] to collect information about technology acceptance, and the SUS for perceived usability [20].

### Statistical Analysis

Data analysis was performed using IBM SPSS Statistics (version 26; IBM Corp), and JASP (version 0.11.1). Descriptive statistics (mean, median, standard deviation, minimum, maximum, skewness, and kurtosis) were calculated for each variable. Normality was considered adequate if absolute values for skewness and kurtosis were above 2.0 and 7.0, respectively [39,40].

### Structural Validity

The creators of the original scale analyzed the factor structure through principal component analysis (PCA) [23]. Results indicated two components with an eigenvalue greater than 1. The ﬁrst component had all six items and explained 43% of the variance, and the second component had only four items (items 1, 4, 5, and 6), only two of which had factor loadings greater than 0.5. Therefore, after the analysis of the scree plot, they considered a one-factor solution explaining 42.9% of the variance to be appropriate.

Before conducting exploratory factor analysis (EFA) with our data, the “parameters” R package was used to decide the number of factors to extract. The solution for one dimension was supported by 6 (42.9%) methods of 14 (acceleration factor, standard error scree, Tucker-Lewis index [TLI], root mean square error of approximation [RMSEA], adjusted root mean square residual, and Bayesian information criterion) [41]. As PCA is a data reduction technique that does not conduct to a latent variable model, principal axis factoring was used to extract the latent factor.

Variables with factor loadings above 0.4 were extracted. Before the analysis, the appropriateness of the data for factor analysis was examined using the Kaiser-Meyer-Olkin (KMO) measure of sampling adequacy and Bartlett test of sphericity [42,43]. Regarding the sample size for the EFA, there is no consensus on the number of participants required to perform the analysis [44,45]. Hatcher et al [46] recommend a minimum subject to item ratio of at least 5:1, with a minimum of 100 subjects. The number of components was determined by Kaiser criteria (retaining factors with eigenvalues greater than 1), scree plot inspection [43], and parallel analysis [47].

Subsequently, confirmatory factor analysis (CFA) was performed using maximum likelihood estimation. The factor loadings were used as local indices of goodness of ﬁt as well as the following goodness-of-fit indices and thresholds for a good fit: chi-square test (χ^2^; *P*>.05), chi-square value divided by degrees of freedom (a ratio of ≤3), comparative fit index (CFI≥0.90), Tucker-Lewis index (TLI≥0.90), goodness of fit index (GFI ≥0.90), root mean square error of approximation (RMSEA<0.08) and standardized root mean square residual (SRMR≤0.08) [48-50].

Ideally, a CFA should be performed in a subsequent study using another sample (validation sample) [51]. However, due to having an insufficient sample size to perform the analysis in two subsamples, the same sample was used for both approaches (EFA and CFA).

### Internal Consistency

Internal consistency was assessed using both Cronbach α and McDonald ω coefficients [52,53]. Cronbach α is the most widely used measure of reliability. However, it overestimates the true composite reliability and is negatively biased when used to measure the reliability of ordinal variables [52,54]. Given the ordinal response format of USEQ items, McDonald ω was used to overcome these limitations, providing a more accurate approximation of the scale reliability [55]. Cronbach α and McDonald ω coefficients over .70 are considered indicators of satisfactory item homogeneity [55,56]. Item-total and inter-item correlations were analyzed, considering cutoff values over .30 and under .70, respectively [57].

### Construct Validity: Convergent and Divergent Validity

Convergent and divergent validity were estimated using Spearman correlation (*r*). It was hypothesized a priori that the USEQ score would be positively correlated with the SUS score and TAM 3 score, while correlation was not expected with the MMSE score. A coefficient of *r*=0.3 is assumed to provide evidence of convergent and divergent validity [58,59].

### USEQ Scores

Descriptive statistics and normality were assessed for the USEQ’s total score. Correlations between USEQ scores and demographic as well as mood and global cognitive characteristics were estimated using Spearman correlation.

### Ethics Statement

The study was conducted in accordance with the Declaration of Helsinki and was approved by the local and national ethics committees (approval number 42-2018). The study goals and assessments were explained to potential participants. All participants provided written informed consent before study enrollment.

## Results

### Study Participants

A total of 110 participants completed the USEQ questionnaire. Demographic, mood, and global cognitive characteristics of the sample are presented in Table 1. Participants had an average age of 68.41 (SD 3.11) years with a mean of 7.95 (SD 5.38) years of education.

**Table 1:** Characteristics of the study participants (N=110).

Characteristics	Values
Age, years, mean (SD)	68.41 (3.11)
Gender, male, n (%)	50 (45.5)
Education, years of formal schooling, mean (SD)	7.95 (5.38)
Mini-Mental State Examination, total score, mean (SD)	26.95 (2.00)
Geriatric Depression Scale, total score, mean (SD)	6.05 (4.58)

Study participants responded to all items of the USEQ. Descriptive statistics for USEQ items are presented in Table 2. Given the ordinal nature of the variables assessed, item distribution demonstrates some degree of nonnormality. In fact, most data collected in behavioral research does not follow univariate normal distributions [40,60]. For the USEQ total score, the results reveal acceptable values for both skewness (sk=–2.02) and kurtosis (k=3.96), showing no severe violation of normality. Regarding the individual items, the kurtosis value was not acceptable for item 1; thus, it was excluded from further analysis.

**Table 2:** Descriptive statistics for User Satisfaction Evaluation Questionnaire items.

Items	Minimum	Maximum	Median	Mean (SD)	Skewness	Kurtosis
Item 1	1	5	5	4.80 (0.59)	–3.81	17.67
Item 2	3	5	5	4.82 (0.47)	–2.66	6.46
Item 3	2	5	5	4.65 (0.71)	–2.21	4.50
Item 4	2	5	5	4.47 (0.75)	–1.30	0.98
Item 5	1	5	5	4.65 (0.93)	–2.71	6.15
Item 6	1	5	5	4.70 (0.69)	2.68	8.30

### Structural Validity

An exploratory factor analysis was conducted on the 5 items of the USEQ. The Kaiser-Meyer-Olkin measure demonstrated adequacy for the analysis (KMO=0.629) as a value above 0.6 indicates an adequate sample size [43]. The Bartlett sphericity test (χ^2^_10_=126, *P*<.001) indicated that the correlation between the items is sufficient to perform the analysis. The analysis showed that the one-factor solution explains 47% of the variance (Table 3) and comprises all items with factor loadings higher than 0.3. Our findings corroborated the decision of the original questionnaire authors, who considered a one-factor solution to be appropriate.

**Table 3:** Factor matrix containing obliquely unrotated factor loadings of principal axis factoring (forcing one-factor solution). The eigenvalue and the percentage of variance explained by the factor are also shown.

Items	Factor 1
Item 2	0.568
Item 3	0.870
Item 4	0.680
Item 5	0.403
Item 6	0.346
Eigenvalue	2.345
Percent of variance	46.9

**Figure 1:**
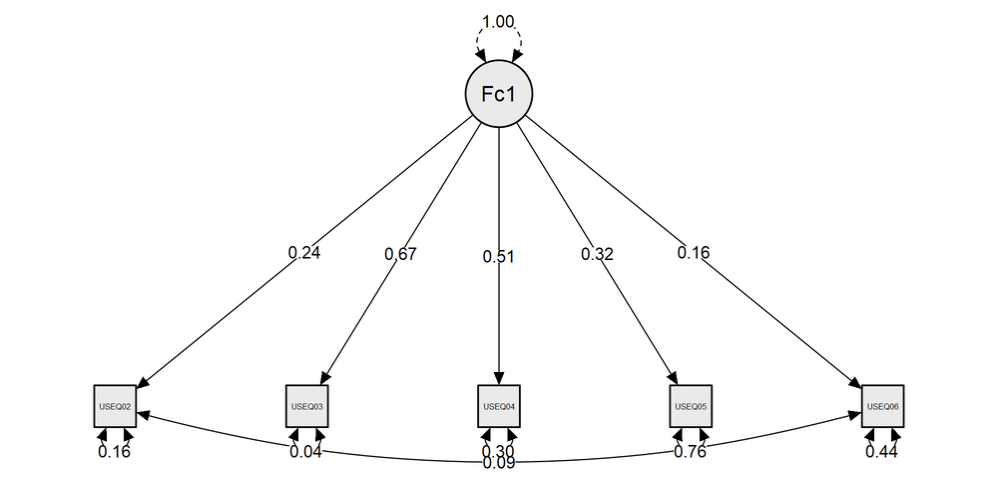
Path diagram and standardized estimates for the one-factor model of the User Satisfaction Evaluation Questionnaire.

A one-factor solution CFA model was tested for the interference dimension, as hypothesized by the original authors [23]. All 5 items were loaded onto a single latent variable. Table 4 shows goodness-of-fit measures for the model, revealing acceptable measures for the following indices: χ^2^_4_=7.313, *P*=.12; CFI=0.973, TLI=0.931, GFI=0.977, RMSEA=0.087, and SRMR=0.038 [61]. The ﬁnal model is presented in Figure 1.

**Table 4:** Fit indices for confirmatory factor analysis model.

Indices	Model
Chi-square value (df)	7.313 (4)
Chi-square value to df ratio	1.83
*P* value	.12
Comparative fit index	0.973
Tucker-Lewis index	0.931
Goodness of fit index	0.977
Root mean square error of approximation	0.087
Standardized root mean square residual	0.038

### Internal Consistency

Analysis of the internal consistency showed acceptable reliability (Cronbach α=.677; McDonald ω=0.722), indicating a reliability homogeneity of the items for the one-factor solution. The corrected item-total correlation values ranged from 0.080 to 0.654, showing an adequate correlation of each item and suggesting adequate scale homogeneity. The inter-item correlations were all below 0.70, indicating nonredundancy of items (Table 5).

**Table 5:** Inter-item correlation matrix.

Items	Item 2	Item 3	Item 4	Item 5
Item 3	0.495	—^a^	—	—
Item 4	0.270	0.654	—	—
Item 5	0.251	0.317	0.327	—
Item 6	0.397	0.219	0.207	0.080

^a^Not applicable.

### Convergent and Divergent Validity

The association between the USEQ and SUS was moderate in magnitude (*r*=0.43, *P*<.001), and strong between TAM 3 factor “perceived ease of use” (*r*=0.690, *P*<.001) and TAM 3 factor “perceptions of external control” (*r*=0.571, *P*<.001). No signiﬁcant correlation was observed between USEQ and MMSE (*r*=0.042, *P*=.661).

### USEQ Scores

To rate the USEQ score, the following classification was used: poor (0-5), fair (5-10), good (10-15), very good (15-20), or excellent (20-25) [62]. The mean USEQ score was 23.30 (SD 2.40), indicating an excellent level of satisfaction with the activity tracker (Xiaomi Mi Band 2; Table 6).

**Table 6:** Scores obtained on the User Satisfaction Evaluation Questionnaire for the original scale with 6 items and the newly proposed scale with 5 items.

Items	Minimum	Maximum	Mean (SD)	Skewness	Kurtosis
USEQ (5 items)	14	25	23.30 (2.40)	–1.81	2.99
USEQ (original)	17	30	28.07 (2.84)	–2.01	3.96

All participants reported user satisfaction experiences above “good,” with 90% of all participants reporting excellent satisfaction with the device. Furthermore, a significant correlation (*r*=–0.319, *P*=.001) between depressive mood and user satisfaction was noted; a higher score on the GDS (ie, higher depressive mood) was negatively associated with satisfaction with the device (Table 7).

**Table 7:** Spearman bivariate correlations between User Satisfaction Evaluation Questionnaire scores and demographic, mood, and global cognitive characteristics.

Values	Gender	Age	Years of education	Mini-Mental State Examination	Geriatric Depression Scale
Spearman correlation coefficient (*r*)	–0.005	–0.120	0.124	0.042	–0.319^a^
*P* value	.96	.21	.20	.66	.001

^a^Significant correlation.

## Discussion

Physical activity is associated with health beneﬁts, a decreased burden of disease, and a decrease in all-cause mortality in adults [2]. Nowadays, wearable activity trackers provide the opportunity to increase physical activity levels through continuous monitoring [8], which may be especially beneficial for older adults. However, over 75% of the over-65 age group state that they require assistance to use new technologies [2]. Usability studies on wearable activity trackers are needed to better understand the barriers that older adults face when using these technologies. In a cohort of older adults, this study aimed to provide a valid questionnaire to evaluate user satisfaction using an activity tracker (Xiaomi Mi Band 2).

The results from the translation phase show that the items were easy to understand and that there were no semantic problems. Moreover, the translated items were considered equivalent to the original version. In particular, the USEQ was found to be a simple and easy-to-understand questionnaire with an appropriate number of questions to apply in older populations. Validity evidence was obtained with 5 questions, maintaining the original one-factor structure. Similar to the original study by Gil-Gómez et al [23], reporting that the one-factor solution explained about 43% of the variance, here an approximate 47% of the variance was explained by the one-factor solution.

Confirmatory factor analysis showed acceptable fit indexes (χ^2^_4_=7.313, *P*=.12, CFI=0.973, TLI=0.931, GFI=0.977, RMSEA=0.087, and SRMR=0.038). However, it is recommended to evaluate structural validity in another independent study. The reliability results show that the European Portuguese version of the USEQ yielded an adequate internal consistency (Cronbach α=.677; McDonald ω=0.722) in a sample of older individuals. Overall, results indicate that the psychometric properties of the European Portuguese version of the USEQ are comparable with those of the original version, and therefore may be used for the evaluation of satisfaction concerning other technologies, including wearable activity trackers.

Convergent validity is one of the fundamental aspects of construct validity and it refers to how closely the new questionnaire is related to other variables and other measures of the same construct. Regarding convergent validity, as expected, the USEQ correlated with the SUS (*r*=0.43, *P*<.001). The SUS is a widely used standardized questionnaire for the assessment of the perceived usability of technology. Thus, a moderate positive correlation was expected, given that the two questionnaires are meant to measure similar constructs. TAM 3 has been one of the most influential models regarding technology acceptance. It distinguishes two concepts influencing an individual’s intention to use new technology: perceived ease of use and perceived usefulness. Therefore, we also expected a correlation between some constructs of the TAM 3 and the USEQ. Indeed, results showed a positive moderate correlation of the USEQ with the TAM 3 factors “perceived ease of use” (*r*=0.690, *P*<.001) and “perceptions of external control” (*r*=0.571, *P*<.001).

No demographic variables were found to be significantly correlated to user satisfaction with the device. However, a higher depressive mood, as evaluated by the GDS, was negatively associated with the satisfaction perceived by participants using the device. Similarly, a recent study investigating the impact of depressive symptoms on measures of web user experience found a significant association between depressive symptoms and subjective user experience [63]. These results indicate that mood may be a factor influencing technology usability and may warrant further guidance and/or targeted approaches in the use of these technologies by specific populations.

Regarding user satisfaction with the Xiaomi Mi Band 2, the device achieved a score of 23.30 (SD 2.40) in the USEQ, demonstrating an excellent reported level of satisfaction and thus suggesting suitability for older adults. Furthermore, results showed that item 4 (“Is the information provided by the system clear?”), which is related to the perceived ease of use, yielded the lowest score. This result may suggest that older adults could have difficulties in understanding the information provided by the activity tracker, which should be noted by manufacturers. The perceived ease of use refers to the degree to which a person perceives how easy it is to use the technology and is one of the primary factors that affect an individual’s intention to use new technology [7,9,31]. This kind of difficulty is especially interesting considering the age of the participants enrolled in the study. Older adults tend to perceive technologies as difficult to use due to usability problems related to poor memory, decreased vision, and poor literacy [64], but this may not necessarily be the case for all older individuals. Thus, results should be interpreted with caution and future studies should include cohorts with different characteristics (for instance, higher school levels or those that have been [early] adopters of different types of technologies).

Concerning limitations, the study was conducted using a convenience sample; therefore, the participants may not represent the entire older population. Moreover, if the sample used in this study is more homogenous than the wider population on the common factors, this can lead to attenuation in correlations and can influence the strength or bias of correlations among variables [51]. Future studies should have a larger sample, and it would be beneficial to the study to maximize variance on measured variables relevant to the constructs of interest [51]. It would also be of value to evaluate the long-term use of this device and motivations for long-term use. Moreover, studies combining quantitative and qualitative methods, such as interviews, would also be valuable to explore older adults’ perceptions and experiences, and to provide details about user behaviors, user needs, and specific problems that quantitative measures cannot address. Finally, further user satisfaction studies of older adults using activity trackers should include other devices. This would ensure that such devices can be effectively implemented in clinical and research settings to promote physical activity.

In conclusion, the European Portuguese version of the USEQ has adequate psychometric properties consistent with the original version, supporting its use as a valid and reliable tool for measuring user satisfaction in older adults. Furthermore, we adapted USEQ to a generic questionnaire for user satisfaction that can be used with several mHealth technologies, including smartphones, patient monitoring devices, tablets, mobile health apps, personal digital assistants, and other wireless devices. Finally, this study has contributed to the currently available and growing body of information on the usability of wearable technologies among older adults.

## Appendix

Multimedia Appendix 1 Translation process of the User Satisfaction Evaluation Questionnaire items.

## Abbreviations

CFA: confirmatory factor analysis
CFI: comparative fit index
EFA: exploratory factor analysis
GDS: Geriatric Depression Scale
GFI: goodness of fit index
KMO: Kaiser-Meyer-Olkin
mHealth: mobile health
MMSE: Mini-Mental State Examination
PCA: principal component analysis
PSSUQ: Post-Study System Usability Questionnaire Usefulness
QUIS: Questionnaire for User Interaction Satisfaction
RMSEA: root mean square error of approximation
SRMR: standardized root mean square residual
SUS: System Usability Scale
TAM 3: technology acceptance model
TLI: Tucker-Lewis index
USE: Satisfaction and Ease of Use
USEQ: User Satisfaction Evaluation Questionnaire
WHO: World Health Organization

## References

[1] Turner-McGrievy G, Jake-Schoffman DE, Singletary C, Wright M, Crimarco A, Wirth MD, Shivappa N, Mandes T, West DS, Wilcox S, Drenowatz C, Hester A, McGrievy MJ (2019). Using Commercial Physical Activity Trackers for Health Promotion Research: Four Case Studies. Health Promot Pract.

[2] Steinert A, Haesner M, Steinhagen-Thiessen E (2017). Activity-tracking devices for older adults: comparison and preferences. Univ Access Inf Soc.

[3] Schlomann A, Seifert A, Rietz C (2019). Relevance of Activity Tracking With Mobile Devices in the Relationship Between Physical Activity Levels and Satisfaction With Physical Fitness in Older Adults: Representative Survey. JMIR Aging.

[4] Melin J, Bonn SE, Pendrill L, Trolle Lagerros Y (2020). A Questionnaire for Assessing User Satisfaction With Mobile Health Apps: Development Using Rasch Measurement Theory. JMIR mHealth uHealth.

[5] El-Amrawy F, Nounou MI (2015). Are Currently Available Wearable Devices for Activity Tracking and Heart Rate Monitoring Accurate, Precise, and Medically Beneficial?. Healthc Inform Res.

[6] Maher C, Ryan J, Ambrosi C, Edney S (2017). Users' experiences of wearable activity trackers: a cross-sectional study. BMC Public Health.

[7] Rupp MA, Michaelis JR, McConnell DS, Smither JA (2018). The role of individual differences on perceptions of wearable fitness device trust, usability, and motivational impact. Appl Ergon.

[8] Kononova A, Li L, Kamp K, Bowen M, Rikard R, Cotten S, Peng W (2019). The Use of Wearable Activity Trackers Among Older Adults: Focus Group Study of Tracker Perceptions, Motivators, and Barriers in the Maintenance Stage of Behavior Change. JMIR mHealth uHealth.

[9] Preusse KC, Mitzner TL, Fausset CB, Rogers WA (2017). Older Adults' Acceptance of Activity Trackers. J Appl Gerontol.

[10] Liang J, Xian D, Liu X, Fu J, Zhang X, Tang B, Lei J (2018). Usability Study of Mainstream Wearable Fitness Devices: Feature Analysis and System Usability Scale Evaluation. JMIR mHealth uHealth.

[11] Bevan N, Carter J, Earthy J, Geis T, Harker S (2016). New ISO Standards for Usability, Usability Reports and Usability Measures. Proceedings, Part I, of the 18th International Conference on Human-Computer Interaction. Theory, Design, Development and Practice.

[12] He Z, Kim S, Gong D (2017). The Influence of Consumer and Product Characteristics on Intention to Repurchase of Smart band Focus on Chinese Consumers. International Journal of Asia Digital Art and Design Association.

[13] Chao C (2019). Factors Determining the Behavioral Intention to Use Mobile Learning: An Application and Extension of the UTAUT Model. Front Psychol.

[14] Harrison R, Flood D, Duce D (2013). Usability of mobile applications: literature review and rationale for a new usability model. J Interact Sci.

[15] Brooke J (1996). SUS: A 'Quick and Dirty' Usability Scale. Usability Evaluation in Industry.

[16] Zhou L, Bao J, Setiawan IMA, Saptono A, Parmanto B (2019). The mHealth App Usability Questionnaire (MAUQ): Development and Validation Study. JMIR mHealth uHealth.

[17] Mohamad Marzuki MF, Yaacob NA, Yaacob NM (2018). Translation, Cross-Cultural Adaptation, and Validation of the Malay Version of the System Usability Scale Questionnaire for the Assessment of Mobile Apps. JMIR Hum Factors.

[18] Lewis JR (1995). IBM computer usability satisfaction questionnaires: Psychometric evaluation and instructions for use. International Journal of Human-Computer Interaction.

[19] Gao M, Kortum P, Oswald F (2018). Psychometric Evaluation of the USE (Usefulness, Satisfaction, and Ease of use) Questionnaire for Reliability and Validity. Proceedings of the Human Factors and Ergonomics Society Annual Meeting.

[20] Martins AI, Rosa AF, Queirós A, Silva A, Rocha NP (2015). European Portuguese Validation of the System Usability Scale (SUS). Procedia Computer Science.

[21] Dantas C, Jegundo A, Quintas J, Martins A.I, Queirós R, Rocha N (2017). European portuguese validation of usefulness, satisfaction and ease of use questionnaire (USE). Recent Advances in Information Systems and Technologies. WorldCIST.

[22] Chin J, Diehl V, Norman K (1988). Development of an instrument measuring user satisfaction of the human-computer interface. CHI '88: Proceedings of the SIGCHI Conference on Human Factors in Computing Systems.

[23] Gil-Gómez JA, Manzano-Hernández P, Albiol-Pérez S, Aula-Valero C, Gil-Gómez H, Lozano-Quilis J (2017). USEQ: A Short Questionnaire for Satisfaction Evaluation of Virtual Rehabilitation Systems. Sensors (Basel).

[24] Seifert A, Schlomann A, Rietz C, Schelling HR (2017). The use of mobile devices for physical activity tracking in older adults' everyday life. Digit Health.

[25] Farina N, Lowry RG (2017). Older adults' satisfaction of wearing consumer-level activity monitors. J Rehabil Assist Technol Eng.

[26] Lenz AS, Gómez Soler I, Dell'Aquilla J, Uribe PM (2017). Translation and Cross-Cultural Adaptation of Assessments for Use in Counseling Research. Measurement and Evaluation in Counseling and Development.

[27] World Health Organization (2007). Process of translation and adaptation of instruments.

[28] Campbell DT, Fiske DW (1959). Convergent and discriminant validation by the multitrait-multimethod matrix. Psychological Bulletin.

[29] Revythi A, Tselios N (2019). Extension of technology acceptance model by using system usability scale to assess behavioral intention to use e-learning. Educ Inf Technol.

[30] Lewis JR (2018). The System Usability Scale: Past, Present, and Future. International Journal of Human–Computer Interaction.

[31] Davis FD (1989). Perceived Usefulness, Perceived Ease of Use, and User Acceptance of Information Technology. MIS Quarterly.

[32] Myrberg K, Hydén LC, Samuelsson C (2020). The mini-mental state examination (MMSE) from a language perspective: an analysis of test interaction. Clin Linguist Phon.

[33] Montag C, Panksepp J (2017). Primary Emotional Systems and Personality: An Evolutionary Perspective. Front Psychol.

[34] Yesavage JA, Brink T, Rose TL, Lum O, Huang V, Adey M, Leirer VO (1982). Development and validation of a geriatric depression screening scale: A preliminary report. Journal of Psychiatric Research.

[35] Guerreiro M, Silva A.P, Botelho M, Leitão O, Castro-Caldas A, Garcia C (1994). Adaptação à população portuguesa da tradução do Mini Mental State Examination (MMSE). Rev Port Neurol.

[36] Puri A, Kim B, Nguyen O, Stolee P, Tung J, Lee J (2017). User Acceptance of Wrist-Worn Activity Trackers Among Community-Dwelling Older Adults: Mixed Method Study. JMIR mHealth uHealth.

[37] Mičková E, Machová K, Daďová K, Svobodová I (2019). Does Dog Ownership Affect Physical Activity, Sleep, and Self-Reported Health in Older Adults?. Int J Environ Res Public Health.

[38] Venkatesh V, Bala H (2008). Technology Acceptance Model 3 and a Research Agenda on Interventions. Decision Sciences.

[39] Kim H (2013). Statistical notes for clinical researchers: assessing normal distribution (2) using skewness and kurtosis. Restor Dent Endod.

[40] Curran PJ, West SG, Finch JF (1996). The robustness of test statistics to nonnormality and specification error in confirmatory factor analysis. Psychological Methods.

[41] Makowski D (2018). The psycho Package: an Efficient and Publishing-Oriented Workflow for Psychological Science. JOSS.

[42] Dziuban C.D, Shirkey E.C (1974). When is a correlation matrix appropriate for factor analysis? Some decision rules. Psychological Bulletin.

[43] Howard MC (2015). A Review of Exploratory Factor Analysis Decisions and Overview of Current Practices: What We Are Doing and How Can We Improve?. International Journal of Human-Computer Interaction.

[44] Kyriazos TA (2018). Applied Psychometrics: Sample Size and Sample Power Considerations in Factor Analysis (EFA, CFA) and SEM in General. PSYCH.

[45] Osborne J, Costello A (2004). Sample size and subject to item ratio in principal components analysis. Practical Assessment, Research, and Evaluation.

[46] O'Rourke N, Hatcher L (2013). Step-by-Step Approach to Using the SAS System for Factor Analysis and Structural Equation Modeling.

[47] Patil VH, Singh SN, Mishra S, Donovan T (2007). Parallel Analysis Engine to Aid Determining Number of Factors to Retain [Computer software].

[48] Hooper D, Coughlan J, Mullen M (2008). Structural Equation Modelling: Guidelines for Determining Model Fit. Electronic Journal of Business Research Methods.

[49] Schermelleh-Engel K, Moosbrugger H, Müller H (2003). Evaluating the Fit of Structural Equation Models: Tests of Significance and Descriptive Goodness-of-Fit Measures. Methods of Psychological Research.

[50] Hu L, Bentler PM (1999). Cutoff criteria for fit indexes in covariance structure analysis: Conventional criteria versus new alternatives. Structural Equation Modeling: A Multidisciplinary Journal.

[51] Fabrigar LR, Wegener DT, MacCallum RC, Strahan EJ (1999). Evaluating the use of exploratory factor analysis in psychological research. Psychological Methods.

[52] Şimşek GG, Noyan F (2013). McDonald's ωt, Cronbach's α, and Generalized θ for Composite Reliability of Common Factors Structures. Communications in Statistics - Simulation and Computation.

[53] Trizano-Hermosilla I, Alvarado JM (2016). Best Alternatives to Cronbach's Alpha Reliability in Realistic Conditions: Congeneric and Asymmetrical Measurements. Front Psychol.

[54] Osborne J, Costello A, Kellow T (2005). Best practices in exploratory factor analysis: four recommendations for getting the most from your analysis. Pract Assess Res Eval.

[55] Gadermann A, Guhn M, Zumbo B (2012). Estimating ordinal reliability for Likert-type and ordinal item response data: A conceptual, empirical, and practical guide. Practical Assessment, Research, and Evaluation.

[56] Cronbach LJ (1951). Coefficient alpha and the internal structure of tests. Psychometrika.

[57] Ferketich S (1991). Focus on psychometrics. Aspects of item analysis. Res Nurs Health.

[58] Cohen J (1992). A power primer. Psychological Bulletin.

[59] Schober P, Boer C, Schwarte LA (2018). Correlation Coefficients. Anesthesia & Analgesia.

[60] Norman G (2010). Likert scales, levels of measurement and the “laws” of statistics. Adv in Health Sci Educ.

[61] Schneider F (2017). Measuring Subjective Movie Evaluation Criteria: Conceptual Foundation, Construction, and Validation of the SMEC Scales. Communication Methods and Measures.

[62] di Palo MT (1997). Rating satisfaction research: Is it poor, fair, good, very good, or excellent?. Arthritis Care & Research.

[63] Thielsch MT, Thielsch C (2018). Depressive symptoms and web user experience. PeerJ.

[64] Li Q, Luximon Y (2018). Understanding Older Adults’ Post-adoption Usage Behavior and Perceptions of Mobile Technology. International Journal of Design.

