# Supplementary material for: European Portuguese Version of the User Satisfaction Evaluation Questionnaire (USEQ): Transcultural Adaptation and Validation Study
Source: JMIR Mhealth Uhealth. 2021 Jun 29;9(6):e19245. doi: 10.2196/19245 (PMC8278297; doi:10.2196/19245)
Supplement: Multimedia Appendix 1 [file mhealth_v9i6e19245_app1.docx]

**Supplementary Table 1.** The translation process of the User Satisfaction Evaluation Questionnaire.

| **English version** | **Forward translation** | **Back translation** |
| --- | --- | --- |
| Did you enjoy your experience with the system? | Foi agradável usar esta tecnologia? | Was it enjoyable to use this technology? |
| Were you successful using the system? | Conseguiu usar com sucesso esta tecnologia? | Were you able to successfully use this technology? |
| Were you able to control the system? | Foi capaz de controlar esta tecnologia? | Were you able to control this technology? |
| Is the information provided by the system clear? | A informação fornecida por esta tecnologia foi clara? | Was the information provided by this technology clear? |
| Did you feel discomfort during your experience with the system? | Sentiu-se desconfortável durante o uso desta tecnologia? | Did you feel uncomfortable while using this technology? |
| Do you think that this system will be helpful for your rehabilitation? | Considera que esta tecnologia será útil na sua reabilitação? | Do you think this technology will be useful in your rehabilitation? |

**Supplementary Table 2**. Preliminary version tested in a pilot study with 20 participants and final European Portuguese version obtained after the pilot study.

| **Preliminary version** | **Final version** |
| --- | --- |
| Foi agradável usar esta tecnologia? | Gostou de usar esta tecnologia? |
| Conseguiu usar com sucesso esta tecnologia? | Foi bem-sucedido a usar esta tecnologia? |
| Foi capaz de controlar esta tecnologia? | Foi capaz de controlar esta tecnologia? |
| A informação fornecida por esta tecnologia foi clara? | A informação fornecida por esta tecnologia foi clara? |
| Sentiu-se desconfortável durante o uso desta tecnologia? | Sentiu-se desconfortável durante o uso desta tecnologia? |
| Considera que esta tecnologia será útil na sua reabilitação? | Considera que esta tecnologia será útil na melhoria da sua saúde? |

**Supplementary Table 3**. Original item vs. corresponding item in English and European Portuguese versions.

| **Spanish – Original version** | **English version** | **European Portuguese final version** |
| --- | --- | --- |
| **Te has divertido com el sistema?** | Did you enjoy your experience with the system? | Gostou de usar esta tecnologia? |
| **Superaste com êxito lo planteado por el sistema?** | Were you successful using the system? | Foi bem-sucedido a usar esta tecnologia? |
| **Has sentido que tenias el control de la situación com el sistema?** | Were you able to control the system? | Foi capaz de controlar esta tecnologia? |
| **Te ha parecido clara la información que te ha dado el sistema?** | Is the information provided by the system clear? | A informação fornecida por esta tecnologia foi clara? |
| **Te has sentido incomodo em algun momento durante el ejercicio?** | Did you feel discomfort during your experience with the system? | Sentiu-se desconfortável durante o uso desta tecnologia? |
| **Crees que este tratamento resultará útil para tu rehabilitacion?** | Do you think that this system will be helpful for your rehabilitation? | Considera que esta tecnologia será útil na melhoria da sua saúde? |
